# Diabetes-Induced Changes in Macrophage Biology Might Lead to Reduced Risk for Abdominal Aortic Aneurysm Development

**DOI:** 10.3390/metabo12020128

**Published:** 2022-01-29

**Authors:** Giulia Chinetti, Joseph Carboni, Joseph Murdaca, Claudine Moratal, Brigitte Sibille, Juliette Raffort, Fabien Lareyre, Elixène Jean Baptiste, Réda Hassen-Khodja, Jaap G. Neels

**Affiliations:** 1 Centre Hospitalier Universitaire (CHU), Institut National de la Santé Et de la Recherche Médicale (INSERM), Centre Méditerranéen de Médecine Moléculaire (C3M), Université Côte d’Azur, 06200 Nice, France; raffort-lareyre.j@chu-nice.fr (J.R.); jean-baptiste.e@chu-nice.fr (E.J.B.); hassen-khodja.r@chu-nice.fr (R.H.-K.); 2 Department of Vascular Surgery, Centre Hospitalier Universitaire (CHU), 06000 Nice, France; carboni.j@chu-nice.fr; 3 Institut National de la Santé Et de la Recherche Médicale (INSERM), Centre Méditerranéen de Médecine Moléculaire (C3M), Université Côte d’Azur, 06200 Nice, France; joseph.murdaca@univ-cotedazur.fr (J.M.); Claudine.MORATAL@univ-cotedazur.fr (C.M.); Brigitte.SIBILLE@univ-cotedazur.fr (B.S.); fabien.lareyre@gmail.com (F.L.); 4 Department of Vascular Surgery, Hospital of Antibes Juan-les-Pins, 06160 Antibes, France

**Keywords:** macrophages, inflammation, metabolism, type 2 diabetes

## Abstract

Type 2 diabetes patients are less likely to develop an abdominal aortic aneurysm (AAA). Since macrophages play a crucial role in AAA development, we hypothesized that this decrease in AAA risk in diabetic patients might be due to diabetes-induced changes in macrophage biology. To test this hypothesis, we treated primary macrophages obtained from healthy human volunteers with serum from non-diabetic vs. diabetic AAA patients and observed differences in extracellular acidification and the expression of genes involved in glycolysis and lipid oxidation. These results suggest an increase in metabolism in macrophages treated with serum from diabetic AAA patients. Since serum samples used did not differ in glucose content, these changes are not likely to be caused by differences in glycemia. Macrophage functions have been shown to be linked to their metabolism. In line with this, our data suggest that this increase in macrophage metabolism is accompanied by a shift towards an anti-inflammatory state. Together, these results support a model where diabetes-induced changes in metabolism in macrophages might lead to a reduced risk for AAA development.

## 1. Introduction

Cardiovascular diseases represent important causes of mortality and morbidity, and rank first of the most frequently occurring diseases. Among them, abdominal aortic aneurysm (AAA), defined as a focal dilatation of the aorta superior to 30 mm in diameter, is an early inflammation process causing degradation of the aorta wall, loss of arterial wall parallelism and progressive dilation until eventual fatal rupture [1], representing a life-threatening disease. AAA is an age-related disorder, and its frequency drastically increases with a rapidly aging population worldwide. Prevention and treatment of this pathology has thus become a major public health issue. However, despite advances in the management of patients, specific drug approaches to treat and limit aneurysm growth are still lacking, and the only available curative therapeutic solution is surgical intervention (open or endovascular surgery). Thus, it is of crucial importance to better understand the cellular/biological mechanism beyond AAA formation/progression to develop novel therapeutic strategies. AAA formation results from a complex process mainly involving the remodeling of the extracellular matrix (ECM), infiltration of inflammatory cells within the aortic wall (particularly monocytes/macrophages), as well as impairment of the vascular smooth muscle cell (SMC) homeostasis and increased local oxidative stress [2].

AAA is most often associated with atherosclerosis. Indeed, these two pathologies share common risk factors (age, male sex, hypertension, smoking, dyslipidemia) as well as physio-pathological mechanisms (macrophage-driven inflammation, ECM remodeling and SMC involvement) [3]. Intriguingly, while type 2 diabetes (T2D) represents a major cardiovascular risk factor, epidemiological studies have reported a negative association between T2D and AAA. Indeed, several clinical studies have demonstrated that in patients with T2D, the frequency of AAA occurrence is less, and their growth is slower compared to non-diabetic (ND) subjects (reviewed in [4]). An inverse association thus exists between T2D and the occurrence and rate of AAA. At last, a negative association was found between diabetes and AAA rupture [5]. While it is difficult to dissociate the intrinsic effect of T2D from those of anti-diabetic treatment, several results support the concept that diabetes, per sé, is the overwhelming variable generating protection to AAA [4,6]. An inverse correlation has been reported between fasting blood glucose and glycated haemoglobin A1c (reflecting long-term glucose concentrations) with AAA diameter [7,8]. Moreover, we have demonstrated that serum concentrations of fructosamine, reflecting the glycaemic status over the preceding 2–3-week period, negatively correlated with the AAA diameter [9]. Indeed, using a targeted proteomic approach, we have also published that T2D AAA patients have a distinct plasma inflammatory profile, compared to ND AAA subjects, with a reduced expression of circulating CCL19 and CCL23 chemokines, as well as an increased expression of TNSF14, a member of the TNFα superfamily [10]. Altogether, these results suggest a protective effect of T2D on AAA formation and the understanding of the mechanisms involved could bring innovative therapeutic strategies for the patients. Among cells involved in AAA, monocytes/macrophages are necessary for the development of the pathology, as demonstrated by the fact that their depletion prevents AAA development in experimental mouse models [11]. Within the aneurysmal tissues, macrophages maintain local inflammation and oxidative stress [2], produce metalloproteases (MMPs) and their tissue inhibitors (TIMPs), are responsible for ECM degradation, and affect SMC phenotype and proliferation [12]. While the presence of inflammation and macrophages alters tissue cell metabolism, reciprocally, metabolism plays a predominant role in immune cell functions [13]. Thus, several emerging pieces of evidence indicate that nutrients and metabolites modulate inflammatory pathways and immune cell phenotypes. By these ways, tissue macrophages can adapt their functional and inflammatory phenotypes in response to cellular metabolic changes or to the general metabolic environment of the tissue. Given their significant involvement in the AAA [14], we believe that these cells could play a central role in the protective effects of T2D toward this pathology. We hypothesized that the observed decrease in AAA risk in diabetic patients might be the consequence of diabetes-induced changes in macrophage biology and functions. To test this hypothesis, primary differentiated macrophages obtained from healthy human volunteers were treated with serum from non-diabetic vs. diabetic AAA patients. Our results indicate that T2D AAA serum leads to macrophage cellular immune-metabolic changes, possibly supporting the inverse relationship between T2D and AAA progression.

## 2. Results

### 2.1. Acidification of Primary Human Macrophage Culture Media by Treatment with Serum from Diabetic AAA Patients

To study the differential effects of serum from diabetic vs. non-diabetic AAA patients on human macrophage biology, we selected serum samples from six non-diabetic and six diabetic AAA patients that were matched for age, body mass index (BMI), AAA diameter, glycemia, and risk factors such as smoking, high blood pressure, and dyslipidemia (Table 1). The diabetic AAA patients all received treatment for diabetes, and one out of six received insulin therapy.

**Figure 1:**
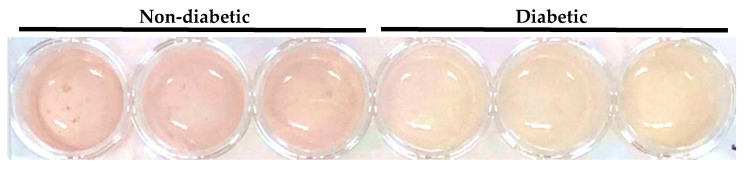
Acidification of primary human macrophage culture media by treatment with serum from diabetic AAA patients. Primary human macrophages cultured in a 24-well plate were treated in triplicate with serum (10% *v/v*) from non-diabetic (left 3 wells) or diabetic (right 3 wells) AAA patients. The difference in acidification is evidenced by the stronger transition to yellow of the pH indicator phenol red present in the media from the cells treated with serum from diabetic AAA patients (right 3 wells).

Primary human monocyte-derived macrophages from healthy volunteers were exposed to these sera (10% *v/v*) for 48 h. To account for donor-related differences, we used cells from a total of seven different donors and each patient serum sample was tested, in triplicate, on macrophage preparations from at least three different healthy donors. We observed that this serum treatment led to differences in acidification of the cell culture media, as visualized by the transition from red to yellow of the pH indicator phenol red present in the media. The media from primary human macrophages treated with 10% (*v/v*) serum from diabetic AAA patients turned yellow, while culture media from the same donor cells remained pink when treated with serum from non-diabetic AAA patients (Figure 1).

### 2.2. Increase in Expression of Genes Involved in Oxidative Phosphorylation and Glycolysis

**Figure 2:**
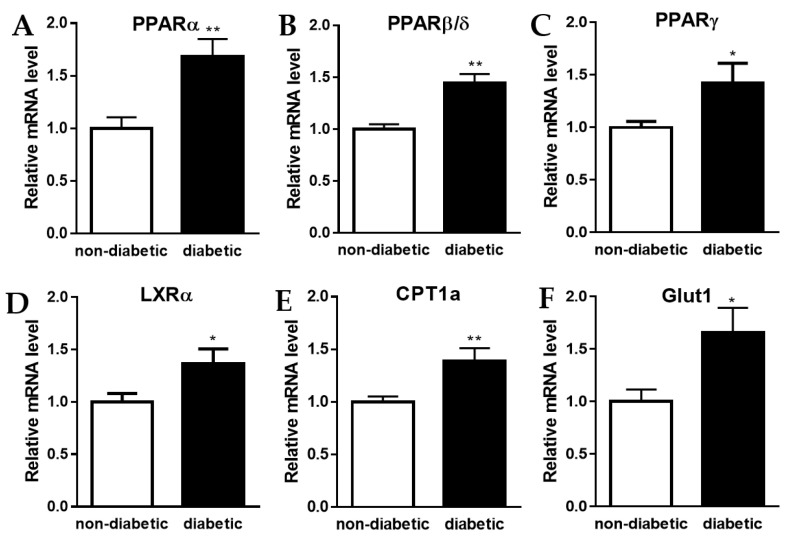
Increase in the expression of genes involved in oxidative phosphorylation and glycolysis. Relative mRNA levels of (**A**) *PPARA*, (**B**) *PPARD*, (**C**) *PPARG*, (**D**) NR1H3 (also known as LXRα), (**E**) CPT1A, and (**F**) SLC2A1 (also known as Glut1) in primary human macrophages that were treated for 48 h with serum (10% *v/v*) from non-diabetic vs. diabetic AAA patients. Each bar represents data from 6 different patient sera tested on cells from eat least 3 different healthy volunteers (7 donors in total) measured in triplicate. Data are shown as mean ± SEM. * *p* < 0.05, ** *p* < 0.01.

Variations in cell culture media acidification are often the consequence of metabolic changes. Lactic acid and CO_2_ produced by glycolysis and substrate oxidation, respectively, are responsible for this extracellular acidification [15]. Peroxisome proliferator-activated receptors (PPARs) are ligand-activated transcription factors of the nuclear receptor (NR) superfamily comprising three subtypes, PPARα, PPARβ/δ, and PPARγ. These PPARs are known for their important roles in regulating the expression of genes involved in cellular metabolism [16]. We show that relative mRNA levels of all three PPAR members are significantly increased in primary human macrophages treated with serum from diabetic compared to non-diabetic AAA patients (Figure 2A–C). Furthermore, the relative mRNA level of another NR that plays an important role in macrophage metabolism, the liver x receptor alpha (LXRα) [17], was similarly increased (Figure 2D). As a measure of lipid oxidation and glycolytic potential, we analyzed the relative mRNA levels of carnitine palmitoyltransferase 1A (CPT1a), the rate-limiting mitochondrial enzyme in fatty acid metabolism, and of the glucose transporter Glut1. Both were significantly increased in the primary human macrophages treated with serum from diabetic AAA patients, compared to cells that were exposed to serum from non-diabetic AAA patients (Figure 2E,F).

### 2.3. Protein Levels of CPT1a and LXRα

**Figure 3:**
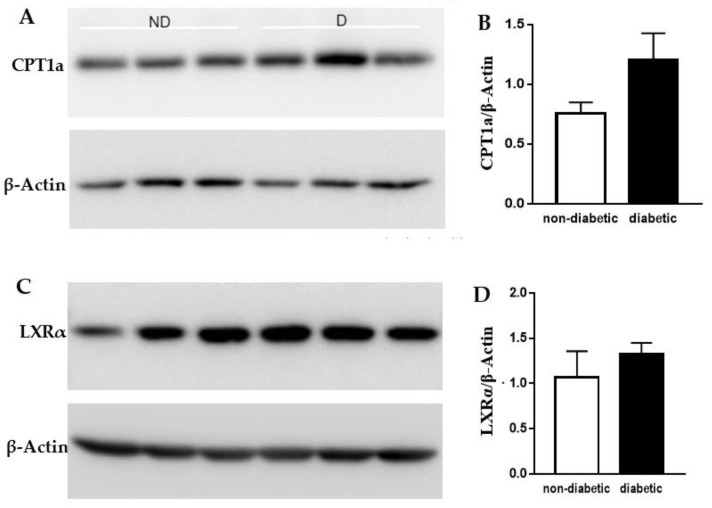
Western blots of CPT1a and LXRα protein levels in primary human macrophages treated with AAA patient sera. Primary human macrophages from a single healthy donor were treated for 48 h with sera (10% *v/v*) from three different non-diabetic (ND) and three different diabetic (D) AAA patients. Western blots of protein lysates stained for (**A**) CPT1a or (**C**) LXRα and β-Actin (after stripping). Quantification of (**B**) CPT1a or (**D**) LXRα signal normalized for β-Actin as loading control. Data in B and D are shown as mean ± SEM.

To confirm that the changes observed at the mRNA level were also occurring at the protein level, we quantified CPT1a and LXRα protein levels by Western blot. Results were normalized to β-Actin levels. As shown in Figure 3, protein levels of both CPT1a and LXRα showed a tendency to be more elevated in primary human macrophages treated with serum from diabetic AAA patients compared to non-diabetic patients, but this increase did not reach statistical significance.

### 2.4. Shift towards an Anti-Inflammatory State

**Figure 4:**
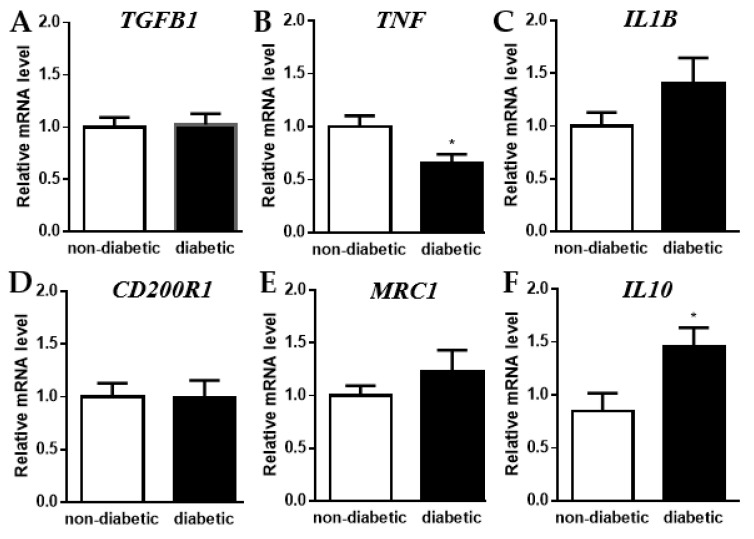
Relative RNA levels of cytokines and macrophage polarization markers in primary human macrophages treated with serum from non-diabetic vs. diabetic AAA patients. Relative mRNA levels measured by qPCR of (**A**) *TGFB1*, (**B**) *TNF*, (**C**) *IL1B*, (**D**) *CD200R1*, (**E**) *MRC1*, and (**F**) *IL10* in primary human macrophages treated for 48 h with serum (10% *v/v*) from non-diabetic vs. diabetic AAA patients. Each bar represents data from 6 different patient sera tested on cells from at least 3 different healthy volunteers (7 donors in total) measured in triplicate. Data are shown as mean ± SEM. * *p* < 0.05.

Changes in macrophage metabolism are often accompanied by a shift in inflammatory state. Therefore, we measured the relative RNA levels of several cytokines and macrophage polarization markers in primary human macrophages treated with serum from non-diabetic vs. diabetic AAA patients. We observed no significant differences in mRNA levels of the anti-inflammatory and pro-fibrotic cytokine TGFβ or the alternative macrophage (M2) marker CD200R1 (Figure 4A,D), a slight but non-significant increase in the pro-inflammatory cytokine IL1β and the M2 macrophage marker MRC1 (Figure 4C,E), a significant decrease in the pro-inflammatory cytokine TNFα (Figure 4B), and a significant increase in the anti-inflammatory cytokine IL10 (Figure 4F). Taken together, these results suggest that the change in macrophage metabolism is accompanied by a shift towards an anti-inflammatory state.

## 3. Discussion

In this study we investigated the differential effects of serum from diabetic vs. non-diabetic AAA patients on human macrophage biology. Our data suggest that primary human macrophages from healthy volunteers exposed to serum from diabetic vs. non-diabetic AAA patients exhibited higher metabolic activity (i.e., both glycolysis and fatty acid oxidation (FAO)), based on increased extracellular acidification and the increased expression of genes/proteins involved in those metabolic pathways (i.e., Glut1, PPARs, LXRα, and CPT1a). Additional experiments are required, such as lactate measurements and/or Seahorse experiments, to investigate the metabolic pathways involved in the observed increase in extracellular acidification. According to their inflammatory status, macrophages are classified as classical activated (pro-inflammatory, M1) and alternatively activated (anti-inflammatory, M2), a classification also associated with distinct metabolic activities [18]. Generally, pro-inflammatory M1 macrophages rely mainly on glycolysis, whereas anti-inflammatory M2 macrophages are more dependent on FAO. However, accumulating evidence suggests that this is an oversimplified model with limited physiological relevance. For example, several recent studies have documented that glycolysis plays a role in M2 activation, and glucose oxidation has been demonstrated to be required for the early differentiation of M2 macrophages [19]. On the other hand, FAO is not essential for M2 activation [19].

Furthermore, M2 macrophages can be further subdivided into M2a, M2b, M2c, and M2d subcategories [20]. Nonetheless, even though the metabolic status of the macrophages might not be foolproof in classifying them according to their inflammatory status, our data on the expression of pro- and anti-inflammatory cytokines and macrophage polarization markers suggest that treatment with serum from diabetic AAA patients shifted the macrophages towards a potential M2b-like state. M2b macrophages typically express increased levels of both IL-1β and IL-10 [21], which we also observed in the macrophages treated with serum from diabetic AAA patients (even though the increase in IL-1β did not reach statistical significance). Furthermore, the expression of CD200R1 (M2a marker), MRC1 (M2a/M2c marker), or TGFβ (prominent in M2c macrophages) did not significantly change in these macrophages. However, M2b macrophages are also known to express TNFα, which was significantly decreased in our serum-treated macrophages, suggesting that these macrophages do not fully share M2b macrophage characteristics.

Interestingly, M2b macrophages express high levels of TNFSF14 [21]. As we mentioned in the introduction, we have previously published that T2D AAA patients have increased levels of TNFSF14 in their plasma compared to non-diabetic AAA patients [10]. Furthermore, another study found that TNFSF14 was increased in the plasma of T2D patients compared to the controls [22], which was concordant with our findings and suggests that this increase is not specific for AAA patients with T2D. Perhaps this increase in plasma TNFSF14 levels in T2D patients is indicative of a shift towards M2b macrophages. In this regard, it is of interest to note that high fat diet-fed obese and insulin-resistant mice were shown to contain M2b macrophages in their adipose and other tissues [23], and similar M2b-like macrophages were identified in human obese adipose tissue as well [24,25]. Together, these findings reinforce the concept that metabolic abnormalities polarize macrophages, whatever their tissular origin, towards M2 macrophages [23].

M2b macrophages are also known as regulatory macrophages because of their immunosuppressive actions, and perhaps this plays a role in reducing the AAA risk in diabetic patients. While both M1 and M2 macrophages were found to be present in aneurysms, studies targeting macrophage differentiation towards the M2 phenotype have been shown to promote the resolution of aortic inflammation and slow AAA progression [26]. M2b macrophage polarization can be induced upon combined exposure to immune complexes and Toll-like receptor (TLR) agonists, or by IL-1R agonists [21]. Perhaps serum from T2D patients is rich in such agonists, or serum from non-diabetics contains inhibitors of M2b macrophage polarization. Future studies are required to identify the factor(s), present or absent in serum from diabetic AAA patients, that are responsible for the changes that we observed in macrophage biology.

Since all the diabetic AAA patients from which we tested the serum were undergoing diabetes treatment, we cannot exclude that the treatment itself played a role in the effects observed. In this regard, it is of interest to note that the use of metformin, the primary oral hypoglycemic agent in use worldwide, was negatively associated with AAA enlargement in patients and dramatically suppressed the formation and progression in an experimental AAA mouse model [27]. Furthermore, metformin was shown to modulate macrophage polarization to the M2 phenotype [28]. The lack of differences in glycemia in our patient groups, most likely due to normalization of glucose levels in the T2D patients by treatment with antihyperglycemic agents, allowed us to conclude that the differences in serum effects observed were not due to differences in glucose levels. However, we cannot exclude that the diabetic patients were not hyperglycemic before, which could have led to the presence of glycated proteins in the serum tested. Glycated hemoglobin (HbA1c) levels reflecting this were not available for the included patients. Since glycated proteins have been shown to modulate the activity of macrophages by activating RAGE (receptor for advanced glycation end products) [29], the differences observed might be the result of these effects.

In vivo and/or ex vivo studies are required to explore whether our in vitro observations are relevant for the pathophysiological events occurring during AAA development. To our knowledge, the presence and role of M2b macrophages in AAA has not been studied, nor has a potential increase in this macrophage subset in diabetic AAA patients. Taken together, we belief that modulation of the macrophage subset polarization, potentially through metabolic intervention, could be an attractive strategy to prevent the development and/or progression of AAA.

## 4. Materials and Methods

### 4.1. AAA Patient Serum Samples

Patients with AAA were included in the Department of Vascular Surgery at the University Hospital of Nice. The study was conducted in conformity with the Declaration of Helsinki and was approved by the institutional local ethics committee. All patients were informed and gave written consent. Inclusion criteria were men older than 18 years with AAA. Aneurysms caused by specific etiologies, including infectious, genetic, or autoimmune diseases, were excluded. Patients receiving anti-inflammatory and immunosuppressive treatments were also excluded. Consecutive type 2 diabetic patients and age- and sex-matched nondiabetic patients were included according to the selection criteria. Preoperative characteristics were collected including age, sex, the presence of cardiovascular risk factors, treatments, and AAA diameter. Type 2 diabetes (T2D) was defined based on medical records and the use of antidiabetic drugs. All diabetic patients were treated with oral antidiabetic drugs, and one required insulin therapy. Nondiabetic patients were defined as patients without any history of diabetes, or the use of antidiabetic drugs. Data were collected using manuscript and electronic medical records as well as a computer software program (Clinicom^®^, Dothan, AL, USA). Blood samples were obtained after a peripheral vein puncture after 8 to 12 h fasting in vacutainer SST II tubes. Serum samples were obtained by centrifugation after collection at room temperature for 10 min at 2200 rpm and were stored at −80 °C.

### 4.2. Primary Human Macrophage Cultures

Human buffy coats from healthy donors (Établissement Français du Sang, Marseille, France) were used to collect PBMCs by FICOLL density gradient centrifugation (human PANCOLL, Pan Biotech, Aidenbach, Germany) [30]. PBMCs were resuspended in RPMI 1640 cell culture media and incubated for 2 h on Primaria 24-well cell culture plates to allow for the adherence of monocytes. Other cell populations were removed by two PBS washes and remaining adhered monocytes were allowed to differentiate into macrophages in RPMI 1640 media supplemented with 10% human commercial serum and gentamycin. After six days of differentiation, the macrophages were washed twice with PBS and then incubated for 48 h with RPMI 1640 supplemented with 10% serum from either non-diabetic or diabetic AAA patients. Serum from a total of 6 non-diabetic and 6 diabetic AAA patients was tested and serum from each patient was used to treat primary human macrophages in triplicate from at least 3 different healthy donors (7 donors total).

### 4.3. RNA Extraction and Quantitative Real-Time PCR

Total RNA was extracted from the AAA patient serum-treated primary human macrophages with Trizol reagent (Invitrogen, Waltham, MA, USA). Then, 1 μg of RNA was reverse-transcribed using a QuantiTect Reverse Transcription Kit (Qiagen, Hilden, Germany) on a Q-cycler II. A quantitative PCR was performed using SYBR Premix Ex Taq (Tli RNase H Plus) (Ozyme, Paris, France) on a StepOne machine (Life Technologies, Carlsbad, CA, USA). The relative amount of all mRNAs was calculated using the comparative ΔΔCT method, and cyclophilin A was used as the housekeeping gene. Primer sequences are available upon request.

### 4.4. Protein Extraction and Western Blot Analysis

Primary human macrophage cultures were washed twice with cold PBS before protein extraction using lysis buffer (100 mM sodium fluoride, 2 mM sodium orthovanadate, 10 mM sodium pyrophosphate, 0.5% sodium deoxycholate, 1% Triton X-100, and 1% protease inhibitors (Complete Protease Inhibitor Cocktail, Roche, Basel, Switzerland)). After protein quantification using a Pierce BCA protein assay kit (Thermo Fisher, Dardilly, France), 25 μg of protein extract was separated by 10% SDS-PAGE and transferred to polyvinylidine difluoride (PVDF) membranes (Millipore, Burlington, MA, USA). After protein transfer, the membranes were blocked with 5% BSA followed by overnight incubation at 4 °C with the primary antibodies (mouse monoclonal antibodies against CPT1A (clone 8F6AE9, ab128568, Abcam, Cambridge, UK), LXRα (clone PPZ0412, ab41902, Abcam, Cambridge, UK), or β-Actin (clone 8H10D10, #3700, Cell Signaling, Danvers, MA, USA). This was followed by incubation with HRP-conjugated anti-mouse secondary antibody (Cell Signaling, Danvers, MA, USA). Luminescent revelation was performed using ECL Western Blotting Substrate (Thermo Fisher, Dardilly, France). Images were captured using a Syngene Pxi (Ozyme, GeneSys software, Saint-Cyr-l’Ecole, France) and quantified using GeneTools software from GeneSys.

### 4.5. Statistical Analyses

Group differences were analyzed using nonparametric Mann–Whitney tests for continuous variables and Fisher exact tests for categorical variables. A two-sided *p* value < 0.05 was considered as significant. Statistical analyses were performed using GraphPad Prism software (version 7.00; GraphPad Software, La Jolla, CA, USA).

## Figures and Tables

**Table 1:** Patient characteristics of serum samples used †.

Characteristic	Non-Diabetics (*n* = 6)	Diabetics (*n* = 6)
Age (years)	70.2 ± 2.4	69.0 ± 2.8
BMI (kg/m^2^)	26.7 ± 3.5	29.1 ± 3.6
Smoking	5/6	5/6
Hypertension	3/6	5/6
Dyslipidemia	2/6	1/6
AAA diameter (mm)	50.8 ± 2.1	50.7 ± 4.7
Symptomatic	4/6	4/6
Glycemia (mmol/L)	7.8 ± 1.4	7.5 ± 1.9
Diabetes treatment	0/6	6/6 ***†
Insulin therapy	0/6	1/6

† Plus-minus values are means ± SD, Five out of the six patients received metformin (biguanide) and one diamicron (sulfonylurea). There were no significant between-group differences in patient characteristics measured, except for diabetes treatment (*** *p* < 0.001). BMI = body mass index.

## Data Availability

The data presented in this study are available on request from the corresponding authors. The data are not publicly available due to the fact that this concerns patient data which, for privacy reasons, is stored on a protected server.
